# Supplementary material for: Assessing the conservation and targets of putative sRNAs in Streptococcus pneumoniae
Source: Microbiol Spectr. 2025 Jun 24;13(8):e03252-24. doi: 10.1128/spectrum.03252-24 (PMC12323649; doi:10.1128/spectrum.03252-24)
Supplement: Supplemental figures and tables — Fig. S1, and Tables S1 and S2. [file spectrum.03252-24-s0008.pdf]

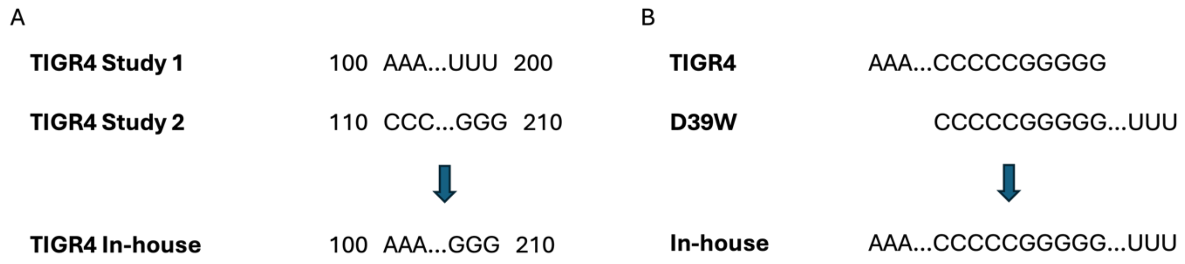

Figure S1: **A)** Formation of the in-house TIGR4 sRNA sequences by combining the sequences identified in the two TIGR4 studies. **B)** Formation of the in-house sRNA sequences by combining the sequences identified in TIGR4 and D39W studies.

	sRNA conservation in other Streptococcus species					
	S. mitis	S. oralis	S. gordonii	S. pyogenes	S. mutans	S. suis
M1						
M4						
M5						
M6						
M7	X	X				
M9	X	X				
M10				X	X	X
M13						
M14	X	X				
M16						
M17						
M18						
M19	X	X	X			
M20	X	X				
M22						
M23						
M24						
M25						
M29	X	X				
M30	X				X	
M32						
M34						
M36		X				
M37						
M39						X
M40		X				
M41						
M42						
M43	X					
M44						X
M45	X					
M46						
M47				X	X	X
M48						
M49						
M50						
M52	X	X				
M53		X				
M59						
M61						
M62				X	X	X
M63	X	X	X	X		
M64						
M65	X	X				
M66						
M67	X	X				
M68	X					
M69				X		
M70		X				
M71						
M73						
M74						
M75						
M76	X	X				
M77	X	X	X	X	X	X
M79						
M80						
M81	X	X	X	X	X	X

Table S1: The conservation of the 58 sRNA candidates in 6 other Streptococcus species. “X” indicates the sRNA in conserved in that species.

sRNA	Target locus	Annotation	Probability	Interaction Length (nt)
M10	SP_RS06190	ISL3 family transposase	0.960	40
M10	SP_RS08360	ISL3 family transposase	0.953	40
M10	SP_RS12115	ISL3 family transposase	0.952	40
M10	SP_RS11735	ISL3 family transposase	0.952	40
M10	SP_RS12420	transposase	0.949	40
M10	SP_RS12405	ISL3 family transposase	0.949	40
M10	SP_RS12565	transposase family protein	0.946	40
M10	SP_RS11780	ISL3 family transposase	0.944	40
M10	SP_RS04090	ISL3-like element IS1167A family transposase	0.941	40
M10	SP_RS05040	ISL3 family transposase	0.941	40
M10	SP_RS11845	transposase	0.934	40
M10	SP_RS00665	ISL3 family transposase	0.930	40
M10	SP_RS02270	ISL3 family transposase	0.929	40
M10	SP_RS11995	ISL3 family transposase	0.926	40
M10	SP_RS08895	ISL3 family transposase	0.925	40
M10	SP_RS08080	ISL3 family transposase	0.924	40
M10	SP_RS07795	ISL3-like element IS1167A family transposase	0.922	40
M10	SP_RS07095	ISL3 family transposase	0.922	40
M47	SP_RS06190	ISL3 family transposase	0.960	39
M47	SP_RS11735	ISL3 family transposase	0.952	39
M47	SP_RS12115	ISL3 family transposase	0.952	39
M47	SP_RS08360	ISL3 family transposase	0.950	39
M47	SP_RS12405	ISL3 family transposase	0.949	39
M47	SP_RS12420	transposase	0.949	39
M47	SP_RS12565	transposase family protein	0.946	39
M47	SP_RS11780	ISL3 family transposase	0.944	39
M47	SP_RS04090	ISL3-like element IS1167A family	0.941	39
M47	SP_RS05040	ISL3 family transposase	0.941	39
M47	SP_RS11845	transposase	0.938	38
M47	SP_RS11995	ISL3 family transposase	0.926	39
M47	SP_RS07095	ISL3 family transposase	0.922	39
M47	SP_RS08080	ISL3 family transposase	0.921	39
M47	SP_RS08895	ISL3 family transposase	0.919	39
M47	SP_RS07795	ISL3-like element IS1167A family	0.916	39
M47	SP_RS00665	ISL3 family transposase	0.722	39
M47	SP_RS02270	ISL3 family transposase	0.710	39
M62	SP_RS11845	transposase	0.734	40

Table S2: The M10, M47, and M62 target predictions in TIGR4 predicted by TargetRNA3. Only the predictions with a probability over 0.7 are shown for each sRNA. M62 only has one predicted transposase target with a probability over 0.7.
